# Office and home blood pressure and their difference according to frailty status among community-dwelling older adults: the NOSE study

**DOI:** 10.1038/s41440-025-02145-8

**Published:** 2025-02-14

**Authors:** Yuka Ohata, Mai Kabayama, Kayo Godai, Michiko Kido, Yaya Li, Yuya Akagi, Naoko Murakami, Hiroko Yoshida, Mariko Hosokawa, Yuka Tachibana, Yuka Fukata, Chihiro Anzai, Kaoru Hatta, Yurie Maeyama, Arisa Wada, Sumiyo Hashimoto, Hiromi Hatanaka, Makiko Higashi, Takeshi Kikuchi, Keiji Terauchi, Fumie Matsuno, Sho Nagayoshi, Kei Asayama, Takayoshi Ohkubo, Hiromi Rakugi, Yasuharu Tabara, Kei Kamide

**Affiliations:** 1 Division of Health Sciences, Graduate School of Medicine, Osaka University, Osaka, Japan; 2 Graduate School of Nursing, Osaka Metropolitan University, Osaka, Japan; 3 Nose Town, Osaka, Japan; 4 OMRON HEALTHCARE Co., Ltd., Kyoto, Japan; 5 Department of Hygiene and Public Health, Teikyo University School of Medicine, Tokyo, Japan; 6 Osaka Rosai Hospital, Osaka, Japan; 7 Graduate School of Public Health, Shizuoka Graduate University of Public Health, Aoi-Ku, Shizuoka Japan

**Keywords:** Community-dwelling older adults, Frailty, Home blood pressure, Office blood pressure, Self-measured

## Abstract

The relationship between frailty and blood pressure (BP) is inconsistent, and limited research has compared BP by frailty status using long-term home BP measurements. We aimed to identify office and home BP and determine differences according to frailty status, stratified by taking antihypertensives in community-dwelling older adults. This cross-sectional study was part of the ongoing non-randomized intervention NOSE study. Participants were aged ≥ 64 years. Frailty was categorized robust, pre-frailty, or frailty using the revised Japanese version of the Cardiovascular Health Study criteria. Office BP was measured in survey settings, and each participant was instructed to take home BP. We used the average home BP for 4 weeks post-survey. An analysis of covariance analyzed the relationship between frailty and office and home BP, and their differences stratified by antihypertensive use. We included 418 older participants (mean age: 72.8 years); 39.5% were male, 40.4% were taking antihypertensives, and 6.7% had frailty. Individuals with frailty taking antihypertensives had higher home morning systolic BP (SBP) than those with robust (134.2 vs. 145.7 mmHg, *P* = 0.018) and pre-frailty (135.6 vs. 145.7 mmHg, *P* = 0.024). The difference between office and morning home SBP in treated participants was 7.1 mmHg (robust), 4.7 mmHg (pre-frailty), and −5.1 mmHg (frailty), showing significant differences (robust vs. frailty: *P* = 0.005, pre-frailty vs. frailty: *P* = 0.016). Home morning SBP was higher in individuals with frailty taking antihypertensives compared to those without frailty, and it may be higher than office BP. Individuals with frailty should measure home BP for good BP control.

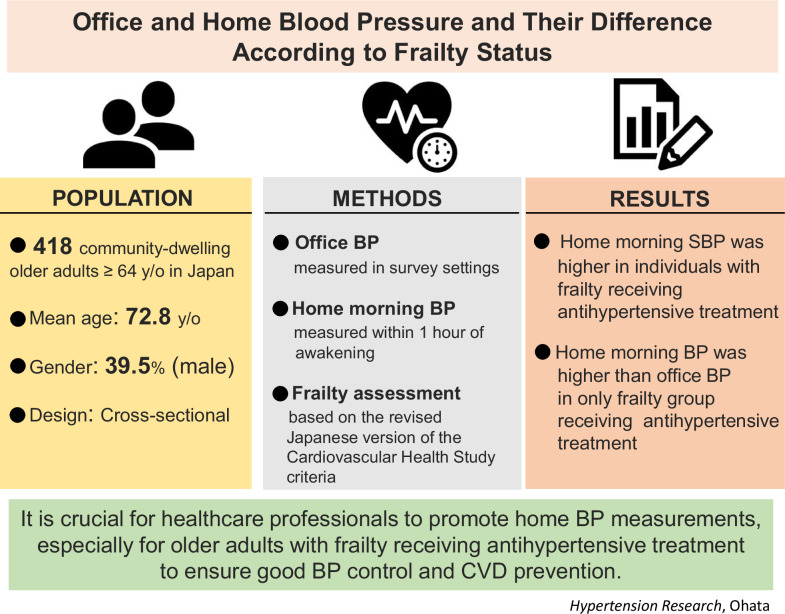

## Introduction

Hypertension is a key risk factor for cardiovascular diseases (CVD) worldwide. Home blood pressure (BP) measurements are crucial for good BP management. Home BP values are more reproducible than office BP values [1]. In addition, healthcare professionals can detect the white-coat effect, morning hypertension, or masked hypertension by reviewing home BP values [2]. Compared to well-controlled hypertension, masked hypertension leads to the progression of atherosclerosis and CVD events [3, 4]. Additionally, home BP measurements are available for untreated individuals and may enable to the early detection of hypertension [5]. Therefore, measuring home BP is considered a useful strategy for maintaining good health status for the entire population [6, 7].

Frailty is commonly seen in older adults, indicating increased vulnerability to stressors and a greater risk of poor health outcomes including disability, hospitalization, and mortality [8, 9]. The relationship between frailty and BP status has shown inconsistent results in previous studies. One study of community-dwelling Japanese older adults (mean age, 68.6 years) reported that individuals with impaired physical function (but not frailty) had higher home BP than those with normal physical function [10]. However, Bastos-Barbosa found no association between frailty and home BP among community-dwelling older adults (mean age: 74.5 years) [11]. In a study of outpatients with cardiometabolic diseases (mean age: 79.2 years), the relationship between frailty and lower office BP was stronger than with higher BP [12]. Similar findings were observed in a cross-sectional study among community-dwelling adults aged 80 years who were taking antihypertensives [13]. Only a few previous studies have evaluated BP using home BP [10, 11] or ABPM [11], whereas the majority relied on office BP measurements [12–16] to investigate the relationship between frailty and BP status.

Maintaining adequate control of morning BP through home BP measurements is very important for CVD prevention [17] because CVD events are more likely to occur in the morning [18]. Morning hypertension, the most common type of masked hypertension, is associated with a higher risk of stroke and need for care in individuals older than 75 years than hypertension defined by office BP value [19, 20]. In Japan, home BP measurements are recommended to be taken upon waking and before bed [6], while office BP values are measured during the day. According to the 2019 Japanese Society of Hypertension Guidelines for the Management of Hypertension (JSH), home BP values are considered more important than office BP values because they are closely associated with CVD events [6]. Moreover, frailty is a risk factors of CVD [21]. Therefore, the relationship between frailty and BP should be investigated using home BP values taken in the morning. However, few studies have investigated this relationship. In addition, identifying the differences between office and home morning BP can ensure more accurate BP control. Previous studies reported greater BP variability in frailty older adults [22], suggesting that office BP may not accurately reflect an individual’s normal BP. As this discrepancy could lead to overtreatment or insufficient BP management, it is necessary to clarify such differences in individuals with frailty. Mori analyzed data stratified by whether participants were receiving antihypertensive treatment [23]. Assessing the association of BP, antihypertensive therapy, and frailty may help us understand these complex interactions and provide insight for BP treatment evidence in the older adults [14]. Furthermore, previous studies investigating the difference between office and home BP measured home BP over 3 days [23] and 2 weeks [24]. Therefore, long-term measurements of home BP, such as over 4 weeks, may provide more accurate BP levels.

The aim of our study was to identify office and home BP and determine differences according to frailty status, stratified by antihypertensive use, among community-dwelling older adults. This study may advance our understanding of the good BP control in older adults and provide clinical and policy implications regarding the need for BP management.

Point of view

- **Clinical relevance:**
  Our findings highlight the importance of promoting home morning BP measurement, particularly among individuals with frailty receiving antihypertensive treatment, to enhance personalized care and improve BP control for CVD prevention.
- **Future direction:**
  Future research should investigate causal relationships through longitudinal studies and develop tailored interventions for improving BP control.
- **Consideration for the Asian population:**
  Given the stronger association between BP and CVD risk and the higher prevalence of morning BP surge in Asians, our study highlights the need to promote home BP monitoring for enhancing BP control for CVD prevention and extending healthy life expectancy.

## Methods

### Study participants

The study participants were recruited from the NOSE study, an ongoing non-randomized intervention study focusing on self-measuring home BP. The main objective of the NOSE study was to determine whether long-term home BP measurements could prevent cognitive decline, frailty, nursing care requirements, and CVD as well as extend the healthy life expectancy. The participants in the NOSE study were recruited between August 2020 and August 2021. We conducted surveys in Nose Town, a regional town with approximately 10,000 inhabitants in Osaka Prefecture. Since the stroke mortality rate is higher in Nose Town than in Osaka Prefecture, a strategy against hypertension is desired. Therefore, the NOSE study was initiated in collaboration with our group and supported by a TOP-Z research grant from OMRON HEALTHCARE Co., Ltd. Participants in the NOSE study were recruited through the town’s newsletter, health checkup settings, community activity sites, and coronavirus disease 2019 vaccination centers. We examined 1,151 community-dwelling adults aged ≥40 years old, representing 15.9% of the Nose Town residents aged at least 40 years. The participants were grouped based on their residential districts into an early intervention group, which started measuring home BP values in 2020, and a late intervention group, which started in 2022. The details of the NOSE study have been published previously [25]. The Ethics Committee of Osaka University Graduate School of Medicine proved the study (approval numbers: 19433-4). Written informed consent was obtained from all participants.

**Fig. 1:**
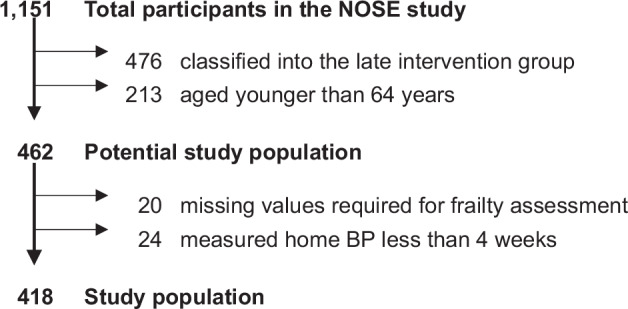
Schematic flow diagram of the selection of study participants

Out of 1151 participants, we excluded 476 allocated to the late intervention group, 213 aged <64 years, 20 with missing data on frailty, and 24 who measured their home BP for less than 4 weeks, resulting in a total of 418 older participants being included. The participant selection method is depicted in Fig. 1. This was a cross-sectional study.

### Definition of frailty

Frailty was determined using the revised Japanese version of the Cardiovascular Health Study (J-CHS) criteria [26], which include weakness, slow walking speed, weight loss, low activity, and exhaustion. Frailty, pre-frailty, and robust were defined by having 3–5, 1–2, and 0 items, respectively. Weakness was defined by grip strength, which was established according to a sex-specific cutoff (<28 kg for men and <18 kg for women). The grip strength of the dominant hand was measured twice using a Smedley hand dynamometer (Model YD-100; Yagami, Ltd., Tokyo, Japan). Measurements were performed in a standing position with the arms resting on the side of the body. Mean values were calculated and used in the analyses. Walking speed was measured over a 5-m walking course at normal speed. Slow walking speed was established using a cutoff <1.0 m/s. Weight loss was assessed using a response of “yes” to the question, “Have you lost 2 kg or more in the past 6 months?” Physical activity was evaluated by asking “Do you engage in moderate levels of physical exercise or sports aimed at health?” and “Do you engage in low levels of physical exercise aimed at health?” If participants answered “no” to both questions, they were classified as having low physical activity. Exhaustion was assessed using a response of “yes” to the following question: “In the past 2 weeks, have you felt tired without a reason?”

### BP measurements

Office BP was measured by medical professionals during the survey and was taken twice consecutively in a sitting position on the right and left upper arms using a portable automated device (HEM-7281T, Omron, Kyoto, Japan). Office BP was defined as the average of four measurements from both arms. Each participant was instructed to take home BP measurements using the same device (HEM-7281T) with the arm that is easier to measure twice a day and record their BP values in their notebooks. Home morning BP was measured twice consecutively in a sitting position within 1 hour of awakening, before taking antihypertensives and breakfast, and after urination, if relevant. Home evening BP was measured before bed. The measurement procedure was in accordance with the recommendations of JSH 2019 [6]. Home BP was determined using the mean of the first and second measurements and the mean home BP levels from all measurements for each participant over a 4-week period after participating in the survey. The difference between office and home BP was calculated as the average of office BP minus the home morning BP for each participant.

### Physical measurements and self-administered questionnaire

Participants were interviewed and physically assessed by physicians, nurses, and physical therapists. Questionnaires were administered to assess the following items: living arrangements, medical history, medication use, smoking habits, and alcohol consumption. Smoking habits were categorized as current smokers or nonsmokers. Those with alcohol consumption habits were classified as regular drinkers. Height, weight, and office BP were measured in the survey settings. Body mass index (BMI) was calculated as weight (kg) divided by height (m) squared. Laboratory data were collected from the medical checkup records (ALT and estimated glomerular filtration rate [eGFR]). Medical history of diabetes, dyslipidemia, stroke, heart disease, and joint disease was assessed using a questionnaire. CVD was defined as individuals who had a stroke or heart disease.

### Statistical analyses

Descriptive statistics are presented as the mean ± standard deviation or frequency. Group differences were assessed by Student’s *t*-test or analysis of variance for continuous variables, while Kruskal-Wallis test was used with non-normal distributions. Frequency differences were assessed by chi-squared test.

An analysis of covariance (ANCOVA) was further performed to clarify whether the differences in morning and evening home BP according to frailty status were independent of possible covariates and to calculate covariate-adjusted means and standard errors. The adjusted factors in the ANCOVA were age, sex, regular drinking, body mass index, history of CVD, and measurement season. Post hoc analysis was performed using Bonferroni’s test. Seasons were defined by the Japan Meteorological Agency as follows: spring from March to May; summer from June to August; autumn from September to November; and winter from December to February. Additionally, we calculated covariate-adjusted mean office and home morning BP differences. Group differences were assessed using ANCOVA after adjusting for age, sex, regular drinking, body mass index, office BP, and measurement season. Post hoc analysis was performed using Bonferroni’s test. Two-tailed *P*-values < 0.05 were considered significant. Statistical analyses were performed using IBM SPSS Statistics version 26 for Windows (IBM Japan, Tokyo, Japan).

## Results

### Characteristics of the participants

Among the 418 older participants, the mean age was 72.8 years old; 39.5% were male, 40.4% were taking antihypertensives. One hundred and twenty-three (29.4%) were classified as robust, 267 (63.9%) as pre-frailty, and 28 (6.7%) as frailty. Table 1 shows the clinical characteristics of the study population according to frailty status. Significant differences in age, regular drinking, and joint disease were found. Supplementary Table 1 represents the class differences in prescribed antihypertensive drugs. Individuals with frailty were more likely to take angiotensin II receptor blockers (ARB) plus calcium channel blockers (CCB) combination therapy and have lower eGFR levels.

**Table 1:** Clinical characteristics of the study participants according to frailty status

		Total	Frailty status			
			Robust	Pre-frailty	Frailty	*P*-value
*N*		418	123	267	28	
Age, years		72.8 ± 5.8	72.1 ± 5.3	72.6 ± 5.6	77.6 ± 7.2	<0.001
Male, %		39.5	43.1	37.5	42.9	0.532
Current smoker, %		5.5	4.1	6.0	7.1	0.685
Regular drinking, %		45.8	54.1	43.8	28.6	0.028
BMI, kg/m^2^		23.4 ± 3.1	23.3 ± 2.7	23.4 ± 3.2	23.5 ± 3.6	0.925
Living alone, %		15.1	11.4	16.9	14.3	0.371
Use of antihypertensives, %		40.4	36.6	40.1	60.7	0.062
Diabetes, %		13.4	13.0	13.5	14.3	0.982
Dyslipidemia, %		39.0	43.1	38.2	28.6	0.330
History of stroke, %		7.9	5.7	8.2	14.8	0.267
History of heart disease, %		19.9	17.1	20.6	25.0	0.561
Joint disease, %		36.2	26.0	39.5	50.0	0.011
Office	SBP, mmHg	137.0 ± 18.4	139.4 ± 18.3	136.0 ± 18.5	134.7 ± 18.1	0.202
	DBP, mmHg	81.0 ± 10.1	82.2 ± 9.2	80.9 ± 10.4	77.3 ± 10.7	0.063
	PR, bpm	73.1 ± 10.9	72.8 ± 10.8	73.3 ± 10.9	74.0 ± 10.9	0.849
Home morning	SBP, mmHg	133.2 ± 15.0	132.5 ± 14.5	133.0 ± 14.5	137.8 ± 20.8	0.223
	DBP, mmHg	80.4 ± 9.3	79.9 ± 8.4	80.6 ± 9.6	80.6 ± 10.4	0.813
	PR, bpm	65.1 ± 8.1	64.2 ± 7.8	65.3 ± 8.1	67.8 ± 9.1	0.098
Home evening	SBP, mmHg	124.5 ± 13.7	124.0 ± 13.0	124.3 ± 13.3	128.2 ± 19.0	0.327
	DBP, mmHg	74.0 ± 8.7	73.3 ± 7.7	74.3 ± 8.8	74.5 ± 11.0	0.600
	PR, bpm	69.2 ± 8.9	69.2 ± 9.1	69.1 ± 8.6	70.1 ± 10.3	0.863

Data are presented as the mean ± standard deviation or frequency. Group differences were assessed by analysis of variance or chi-squared test. *P*-values < 0.05 were considered statistically significant

*BMI* Body mass index, *SBP* Systolic blood pressure, *DBP* Diastolic blood pressure, *PR* Pulse rate

### Office and home BP according to frailty status

**Fig. 2:**
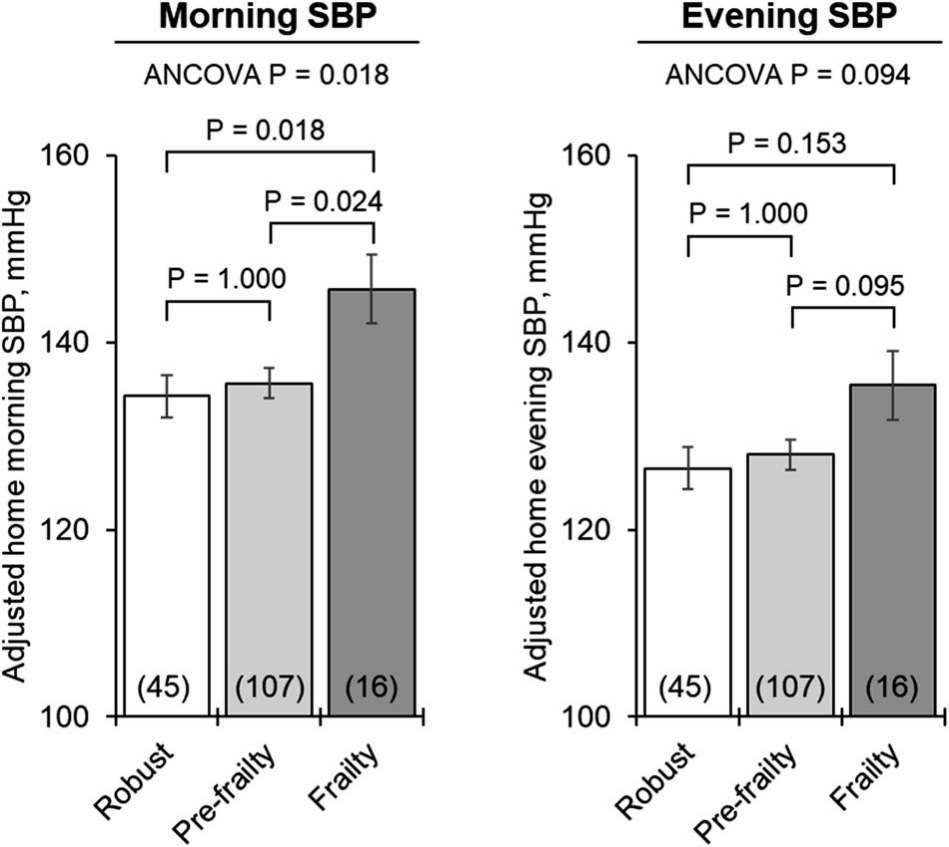
Covariate-adjusted mean home morning and evening SBP according to frailty status in participants receiving antihypertensive treatment. Values are presented as the adjusted mean ± standard error. Group differences were assessed by an analysis of covariance adjusted for age, sex, regular drinking, body mass index, history of cardiovascular disease, and measurement season. Post-hoc analysis was performed using Bonferroni’s test. SBP Systolic blood pressure

Table 2 presents office and home BP measured in the morning and evening according to frailty status, stratified by whether they were treated. A significant difference in the morning home SBP was observed according to frailty status among treated individuals (robust vs. pre-frailty vs. frailty: 134.3 vs. 135.7 vs. 144.8 mmHg, *P* = 0.018). A significant difference in the evening home SBP was also observed among treated individuals (robust vs. pre-frailty vs. frailty: 125.8 vs. 127.2 vs. 135.3 mmHg, *P* = 0.029). No significant differences were found in untreated participants. Figure 2 illustrates the covariate-adjusted mean home morning and evening SBP according to frailty status in participants receiving antihypertensive treatment. Although the home morning SBP was higher in the frailty group (robust vs. frailty: 134.2 vs. 145.7 mmHg, *P* = 0.018; pre-frailty vs. frailty:135.6 vs. 145.7 mmHg, *P* = 0.024), home evening SBP did not show significant differences (robust vs. frailty:126.6 vs. 135.4 mmHg, *P* = 0.153; pre-frailty vs. frailty: 128.1 vs. 135.4 mmHg, *P* = 0.095). No significant differences were found in DBP according to frailty status.

**Table 2:** Differences in office and home BP according to frailty status

			Total	Frailty status			
				Robust	Pre-frailty	Frailty	*P*-value
***Treated***		*N*	169	45	107	17	
	Office	SBP, mmHg	140.7 ± 17.7	144.2 ± 19.6	139.9 ± 16.6	136.8 ± 18.9	0.241
		DBP, mmHg	81.7 ± 10.2	82.8 ± 8.9	81.8 ± 10.5	77.9 ± 11.6	0.239
		PR, bpm	74.1 ± 11.2	76.5 ± 11.4	73.1 ± 11.0	74.0 ± 11.3	0.233
	Home morning	SBP, mmHg	136.2 ± 13.4	134.3 ± 12.5	135.7 ± 11.8	144.8 ± 21.1	0.018
		DBP, mmHg	81.0 ± 9.3	79.6 ± 8.5	81.2 ± 9.4	84.0 ± 10.9	0.238
		PR, bpm	65.5 ± 8.6	65.2 ± 7.4	65.2 ± 8.8	68.2 ± 10.7	0.420
	Home evening	SBP, mmHg	127.7 ± 12.9	125.8 ± 11.3	127.2 ± 11.9	135.3 ± 19.2	0.029
		DBP, mmHg	74.5 ± 8.9	72.8 ± 7.4	74.6 ± 8.9	78.3 ± 11.9	0.099
		PR, bpm	69.8 ± 9.3	70.8 ± 9.1	69.5 ± 9.3	69.2 ± 10.8	0.716
***Untreated***		*N*	249	78	160	11	
	Office	SBP, mmHg	134.4 ± 17.2	136.6 ± 17.0	133.5 ± 19.2	131.5 ± 17.2	0.419
		DBP, mmHg	80.6 ± 10.0	81.9 ± 9.4	80.3 ± 10.4	76.4 ± 9.6	0.187
		PR, bpm	72.5 ± 10.7	70.6 ± 9.9	73.4 ± 10.9	74.0 ± 10.6	0.166
	Home morning	SBP, mmHg	131.0 ± 15.7	131.4 ± 15.6	131.2 ± 15.8	127.2 ± 15.9	0.695
		DBP, mmHg	79.9 ± 9.3	80.1 ± 8.4	80.2 ± 9.8	75.3 ± 7.2	0.235
		PR, bpm	64.9 ± 7.7	63.6 ± 8.0	65.3 ± 7.6	67.2 ± 6.4	0.170
	Home evening	SBP, mmHg	122.3 ± 13.8	123.0 ± 13.8	122.3 ± 13.9	117.2 ± 12.8	0.425
		DBP, mmHg	73.7 ± 9.0	73.6 ± 7.9	74.0 ± 8.8	68.6 ± 6.9	0.118
		PR, bpm	68.8 ± 8.5	68.2 ± 9.0	68.9 ± 8.2	71.4 ± 9.8	0.497

Data are shown as the mean ± standard deviation. Group differences were assessed by analysis of variance. *P*-values < 0.05 were considered statistically significant

*SBP* Systolic blood pressure, *DBP* Diastolic blood pressure, *PR* Pulse rate

### Differences between office and home BP according to frailty status

**Fig. 3:**
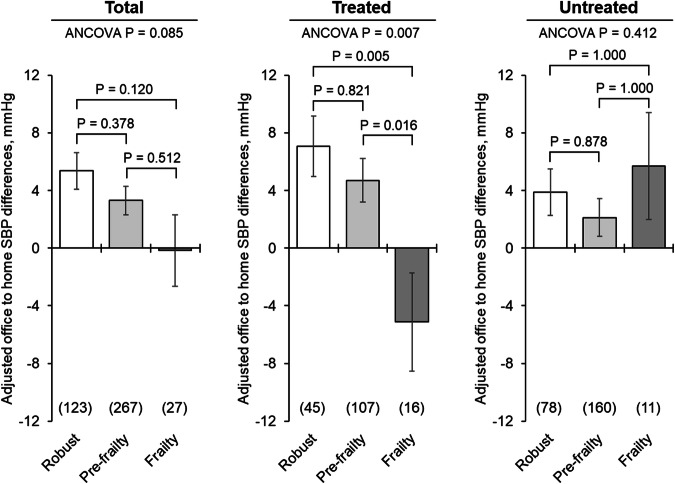
Covariate-adjusted mean office–home morning SBP differences according to frailty and antihypertensive treatment status. Values are presented as the adjusted mean ± standard error. Differences in SBP were calculated by subtracting the home morning SBP from the office SBP. Group differences were assessed by an analysis of covariance adjusted for age, sex, regular drinking, body mass index, office SBP, and measurement season. Post-hoc analysis was performed using Bonferroni’s test. SBP Systolic blood pressure

**Fig. 4:**
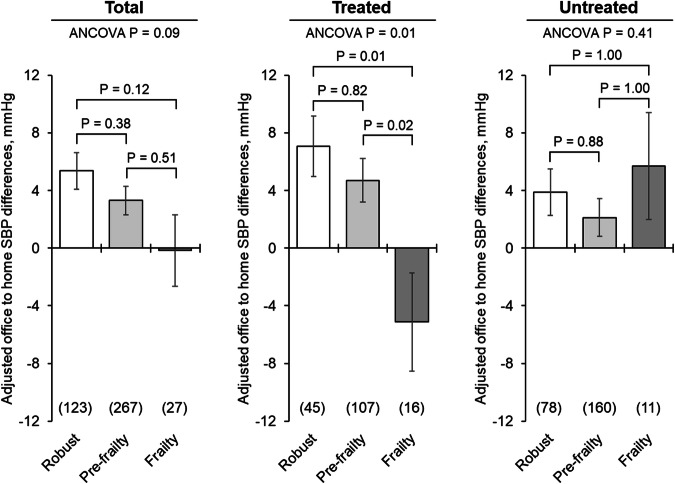
Covariate-adjusted mean office–home morning DBP differences according to frailty and antihypertensive treatment status. Values are presented as the adjusted mean ± standard error. Differences in DBP were calculated by subtracting the home morning DBP from the office DBP. Group differences were assessed by an analysis of covariance adjusted for age, sex, regular drinking, body mass index, office DBP, and measurement season. Post-hoc analysis was performed using Bonferroni’s test. DBP Diastolic blood pressure

Figures 3 and 4 show covariate-adjusted mean office minus home morning BP differences according to frailty and antihypertensive treatment status. The SBP difference among treated participants was 7.1 mmHg in the robust group, 4.7 mmHg in the pre-frailty group, and −5.1 mmHg in the frailty group, showing significant differences (robust vs. frailty: *P* = 0.005, pre-frailty vs. frailty: *P* = 0.016). No significant differences were found in untreated participants (robust vs. frailty: 3.9 vs. 5.7 mmHg, *P* = 1.000; pre-frailty vs. frailty: 2.1 vs. 5.7 mmHg, *P* = 1.000). For DBP in treated participants, the differences were 2.7 mmHg (robust), 0.5 mmHg (pre-frailty), and −5.5 mmHg (frailty), with significant differences among frailty status (robust vs. frailty: *P* = 0.003, pre-frailty vs. frailty: *P* = 0.021).

### Comparison of office and home BP according to frailty

Supplementary Table 2 shows the comparison of office and home BP according to frailty subitems: slow walking speed, weak grip strength, weight loss, exhaustion, and low activity. Home morning SBP was significantly higher in the slow walking speed group (slow vs. normal: 141.0 vs. 132.9 mmHg, *P* = 0.034). Office DBP was significantly lower in weak grip strength group (weak vs. normal: 77.5 vs. 81.6 mmHg, *P* = 0.004).

## Discussion

The home morning SBP was higher in the frailty group than in the robust and pre-frailty groups in individuals receiving antihypertensive treatment. The difference between office and home morning BP tended to vary according to frailty status. In treated participants, office BP was higher than home morning BP in the robust and pre-frailty groups, whereas office BP was lower than home morning BP in the frailty group. In untreated participants, office BP was higher than home BP in all groups. In the sub-analysis, the home morning SBP was higher in the slow walking speed group. Office DBP was lower in the weak grip strength group. This is the first study to identify office and home BP and determine differences according to frailty status among community-dwelling older adults.

In this study, the prevalence of frailty was 6.7%, which is consistent with a previous study on the Japanese general population, suggesting that the study participants are representative of the older Japanese community [27].

Individuals with frailty who took antihypertensives had higher home morning SBP. This finding contrasts with those of previous studies that identified a relationship between frailty and a lower office SBP [12–14]. This may be attributed to differences in the timing of BP measurements. In our study, morning home BP was recorded within 1 hour of awakening. Given that treated individuals with frailty may exhibit an elevated morning SBP, monitoring home BP is particularly important for older adults with frailty to ensure good BP control and CVD prevention.

Regarding the mechanisms underlying an elevated home morning SBP among individuals with frailty, frailty may experience morning or nocturnal hypertension. Frailty had higher BP during sleep compared to non-frailty individuals [11, 28]. Heart failure and poor renal function, which are risk factors for frailty [29, 30], can induce nocturnal hypertension [6]. In our study, individuals with frailty receiving antihypertensive therapy exhibited lower renal function (Supplementary Table 1), which could have influenced their home morning BP values.

The relationship between frailty and differences (office BP minus home morning BP) differed according to frailty status and whether participants were taking antihypertensives, even after controlling for covariates. Office BP was higher than home morning BP among treated participants without frailty, whereas those with frailty had a higher morning BP than office BP. This cannot be ascertained by measuring office BP alone, as it may lead to inadequate hypertension treatment and CVD onset or progression. Thus, we highlight the importance of home BP monitoring in older adults, particularly those with frailty who are undergoing antihypertensive therapy.

The higher office vs. home BP can be explained by the white-coat effect [31]. This phenomenon may not have been observed among participants with frailty, possibly because of antihypertensive-use and dosages. Individuals with frailty were more likely to take them after breakfast (94.1% in frailty vs. 80% in robust group) and more often took ARBs and CCBs in combination (Supplementary Table 1). Therefore, in the frailty group, it is likely that morning home BP was higher because it was measured before medication intake, whereas the medication had taken effect by the time office BP was measured. In addition, the effects of antihypertensive medications may be stronger among participants with frailty. Physiological changes that lead to pharmacokinetic changes such as decreased drug clearance in the liver and kidneys are more likely to occur in the setting of frailty [32]. If the morning home BP was higher than office BP in the frailty group owing to the effects of antihypertensive medication, it is reasonable that office BP was higher than home BP in the untreated frailty group.

In the sub-analysis, home morning SBP was significantly higher in the slow walking speed group, whereas office DBP was significantly lower in the weak grip strength group. A study of community-dwelling older adult participants in the Framingham Heart Study reported that higher levels of frailty were associated with higher levels of arterial stiffness [33]. Arterial stiffness, which occurs when arterial elasticity is diminished, leads to increased blood vessel rigidity, resulting in widened pulse pressure [34, 35]. Consequently, it can lead to higher SBP and lower DBP levels. This result indicates that arterial stiffness may be reflected in BP among older individuals with a slow walking speed or weak grip strength.

This study has some limitations. First, home BP readings were handwritten in notebooks and may contain inaccuracies, leading to measurement bias. Second, healthy, cognitively well older individuals may continue to measure home BP for some time, introducing selection bias. Third, the number of participants with frailty was small. Fourth, we were unable to consider the number and class of antihypertensive medications. Finally, the causal relationship is unclear because of the cross-sectional design. A longitudinal study is needed to identify the causal relationship.

### Perspective of Asia

BP management in Asia has some challenges [36]. The association between BP and the risk of CVD is stronger in Asians than in Western populations [37], especially elevated BP is higher risk for the stroke that is the major cause for long-term care in Asians than in in Western populations [6], and morning BP surge is more commonly observed in Asians [38]. These factors emphasize the critical need for Asians to monitor home BP, especially morning BP, as part of efforts to prevent CVD. Our findings highlight the clinical relevance of home morning BP, particularly in older adults with frailty receiving antihypertensive treatment. Promoting home BP monitoring in Asia is essential for addressing these unique challenges, improving BP control for effective CVD prevention, and extending healthy life expectancy.

## Conclusion

Home morning SBP was higher in individuals with frailty who were undergoing antihypertensive treatment compared to those without frailty. The difference between office and home morning BP varied according to frailty status, suggesting that home morning BP may be higher than office BP in treated individuals with frailty. It is crucial for healthcare professionals to promote home BP measurement, especially among older adults with frailty receiving antihypertensive therapy. This approach allows for personalized and responsive care tailored to each patient’s unique needs—such as the adjustment of antihypertensive medications and guidance on lifestyle factors, including diet and exercise—and contributes to good BP control and CVD prevention.

## Supplementary information

Supplementary Table 1

Supplementary Table 2
