# Supplementary material for: Office and home blood pressure and their difference according to frailty status among community-dwelling older adults: the NOSE study
Source: Hypertens Res. 2025 Feb 14;48(4):1389–98. doi: 10.1038/s41440-025-02145-8 (PMC11972957; doi:10.1038/s41440-025-02145-8)
Supplement: Supplementary file 1 — Supplementary Table 1 [file 41440_2025_2145_MOESM1_ESM.doc]

**Supplementary Table 1**. Class differences in prescribed antihypertensive drugs

|  | Total | Frailty status | | | |
| --- | --- | --- | --- | --- | --- |
|  |  | Robust | Pre-frailty | Frailty | P- value |
| N | 169 | 45 | 107 | 17 |  |
| CCB, % | 60.9 | 73.3 | 56.1 | 58.8 | 0.074 |
| ARB, % | 40.4 | 53.3 | 37.4 | 35.3 | 0.450 |
| ACEI, % | 2.4 | 0 | 3.7 | 0 | 0.317 |
| Diuretics, % | 7.7 | 8.9 | 5.6 | 17.6 | 0.057 |
| α, β, or αβ blocker, % | 9.5 | 8.9 | 3.7 | 6.9 | 0.651 |
| Anti-aldosterone, % | 1.8 | 4.4 | 0.9 | 0 | 0.353 |
| ARB plus diuretics, % | 3.0 | 2.2 | 2.8 | 5.9 | 0.493 |
| ARB plus CCB, % | 10.7 | 2.2 | 12.1 | 23.5 | 0.006 |
| ***Number of prescribed antihypertensive drugs*** |  |  |  |  |  |
| 1, % | 68.6 | 57.8 | 74.8 | 58.8 | 0.109 |
| 2, % | 26.6 | 33.3 | 22.4 | 35.3 |  |
| 3, % | 3.6 | 6.7 | 2.8 | 0 |  |
| 4 or more, % | 1.2 | 2.2 | 0 | 5.9 |  |
| Taking antihypertensives in the morning, % | 82.2 | 80.0 | 81.3 | 94.1 | 0.394 |
| ALT ^1^, IU/L | 17.0 (14.0-22.0) | 17.0 (14.0-24.0) | 18.0 (15.0-22.0) | 15.5 (12.0-20.8) | 0.215 |
| eGFR ^1^, mL/min/1.73m^2^ | 63.1 (55.1-73.5) | 64.5 (51.4-74.0) | 64.4 (56.9-74.0) | 55.6 (49.6-63.0) | 0.031 |

Statistical significance was assessed by chi-square test. ^1^Median (IQR) with the Kruskal–Wallis test

P-values < 0.05 were considered statistically significant.

CCB Calcium channel blockers, ARB Angiotensin II receptor blockers, ACEI Angiotensin-converting enzyme inhibitors
