# Supplementary material for: Office and home blood pressure and their difference according to frailty status among community-dwelling older adults: the NOSE study
Source: Hypertens Res. 2025 Feb 14;48(4):1389–98. doi: 10.1038/s41440-025-02145-8 (PMC11972957; doi:10.1038/s41440-025-02145-8)
Supplement: Supplementary file 2 — Supplementary Table 2 [file 41440_2025_2145_MOESM2_ESM.doc]

**Supplementary Table 2**. Associations between each component of frailty and office and home BP

|  |  |  | Office | | Home morning | | Home evening | |
| --- | --- | --- | --- | --- | --- | --- | --- | --- |
|  |  | N | SBP, mmHg | DBP, mmHg | SBP, mmHg | DBP, mmHg | SBP, mmHg | DBP, mmHg |
| Slow walking speed | yes | 16 | 137.0 ± 16.6 | 79.1 ± 10.1 | 141.0 ± 21.2* | 83.2 ± 10.3 | 133.8 ± 19.7 | 78.3 ± 11.6 |
|  | no | 400 | 137.1 ± 18.5 | 81.1 ± 10.1 | 132.9 ± 14.7 | 80.3 ± 9.2 | 124.1 ± 13.3 | 73.8 ± 8.5 |
| Weak grip strength | yes | 58 | 136.4 ± 20.9 | 77.5 ± 9.3* | 136.0 ± 15.6 | 78.5 ± 9.7 | 127.8 ± 16 | 73.2 ± 9.6 |
|  | no | 358 | 137.0 ± 17.9 | 81.6 ± 10.2 | 132.7 ± 14.8 | 80.6 ± 9.2 | 123.9 ± 13 | 74.0 ± 8.4 |
| Weight loss | yes | 58 | 133.9 ± 16.6 | 78.7 ± 10.2 | 132.0 ± 15.3 | 79.7 ± 9.6 | 123.2 ± 12.8 | 73.0 ± 8.6 |
|  | no | 360 | 137.4 ± 18.7 | 81.4 ± 10.1 | 133.3 ± 15.0 | 80.5 ± 9.3 | 124.7 ± 13.8 | 74.2 ± 8.7 |
| Exhaustion | yes | 96 | 135.0 ± 17.9 | 80.7 ± 11.7 | 134.1 ± 15.9 | 80.8 ± 11 | 125.5 ± 14.9 | 74.4 ± 9.6 |
|  | no | 322 | 137.5 ± 18.5 | 81.1 ± 9.6 | 132.9 ± 14.7 | 80.3 ± 8.8 | 124.2 ± 13.3 | 73.9 ± 8.4 |
| Low activity | yes | 217 | 137.4 ± 17.9 | 81.2 ± 9.8 | 133.1 ± 14.2 | 80.3 ± 9.4 | 125.1 ± 13.4 | 74.0 ± 8.5 |
|  | no | 201 | 136.4 ± 18.9 | 80.9 ± 10.5 | 133.2 ± 15.9 | 80.5 ± 9.3 | 123.8 ± 13.9 | 74.0 ± 8.9 |

Data are presented as the mean ± standard deviation. Group differences were assessed by Student’s t-test. P-values < 0.05 were considered as statistically significant. *P < 0.05

SBP Systolic blood pressure, DBP Diastolic blood pressure
